# Postoperative hypoparathyroidism after thyroid operation and exploration of permanent hypoparathyroidism evaluation

**DOI:** 10.3389/fendo.2023.1182062

**Published:** 2023-05-31

**Authors:** Xi Wang, Shun-lan Wang, Yang Cao, Chun-qiao Li, Weiping He, Zhu-ming Guo

**Affiliations:** ^1^ Department of Otolaryngology, Guangzhou University of Traditional Chinese Medicine First Affiliated Hospital, Guangzhou, China; ^2^ The First School Of Clinical Medicine, Guangzhou University of Chinese Medicine, Guangzhou, China; ^3^ Department of Oncology, Guangzhou University of Traditional Chinese Medicine First Affiliated Hospital, Guangzhou, China; ^4^ Department of Head and Neck, Sun Yat-sen University Cancer Center, Guangzhou, China

**Keywords:** hypoparathyroidism, postoperative complications, thyroid surgery, thyroidectomy, central-compartment neck dissection

## Abstract

**Background:**

To investigate the risk factors for hypoparathyroidism, discuss the prevention of postoperative hypoparathyroidism, and explore permanent postoperative hypoparathyroidism evaluation (PPHE).

**Methods:**

A total of 2,903 patients with thyroid nodules were treated between October 2012 and August 2015. Serum calcium and intact parathyroid hormone (iPTH) levels were measured at 1 day, 1 month, and 6 months postoperatively. The incidence and management of hypoparathyroidism were analyzed. The PPHE was established based on the risk factors and clinical practice.

**Results:**

A total of 637 (21.94%) patients developed hypoparathyroidism, and 92.15% of them had malignant nodules. The incidence rates of transient and permanent hypoparathyroidism were 11.47% and 10.47%, respectively. The iPTH level was lower in patients with malignant nodules who underwent total thyroidectomy (TT) and central-compartment neck dissection (CND). These factors were independently associated with the recovery rate of parathyroid function. The formula for PPHE is as follows: {iPTH} + {sCa} + {surgical procedure} + {reoperation} + {pathologic type}. A scoring system was developed, and we scored low, middle, and high risk of permanent postoperative hypoparathyroidism as 4–6, 7–9, and 10–13, respectively. The differences in the recovery rates of parathyroid function in several risk groups were statistically significant (p < 0.001).

**Conclusion:**

Simultaneous TT and CND is a risk factor for hypoparathyroidism. The reoperation is not associated with hypoparathyroidism. Identification of parathyroid glands *in situ* and preservation of their vascular pedicles are key factors in managing hypoparathyroidism. PPHE can forecast the risk of permanent postoperative hypoparathyroidism well.

## Introduction

Hypoparathyroidism is an abnormality of calcium metabolism characterized by low serum levels of parathyroid hormone leading to hypocalcemia. The most common cause of hypoparathyroidism in adults is postoperative hypoparathyroidism (1), which can lead to hypocalcemia. The symptoms of hypocalcemia can be subtle, such as perioral or digital paresthesia, muscle cramp, or anxiety, but sometimes, it results in serious or life-threatening symptoms such as tetany, cardiac arrhythmia, heart failure, and laryngospasm (2–4); all of these would impact the patient’s quality of life.

Multivariate analysis by various authors confirmed that extent of resection, such as total thyroidectomy (TT) and central-compartment (level VI) neck dissection (CND), is a major risk factor for both transient and permanent hypoparathyroidism after thyroid surgery (5–7). However, there are still some debates on postoperative hypoparathyroidism. Since the risks of postoperative hypoparathyroidism are high when surgeries are performed by low-volume surgeons (fewer than 50 cases/year) (8), can the risks decline when surgeries are performed by experienced surgeons? When conservative initial treatment leads to reoperation, is there a higher risk of postoperative hypoparathyroidism?

The objective of this retrospective analysis was to investigate the risk factors, discuss the prevention of hypoparathyroidism following thyroid surgery in patients with thyroid benign or malignant nodules, and explore permanent postoperative hypoparathyroidism evaluation (PPHE), which can forecast the risk of permanent postoperative hypoparathyroidism.

## Materials and methods

### Patient characteristics

This retrospective analysis enrolled 2,903 patients with thyroid nodules who were admitted to the Sun Yat-Sen University Cancer Center and underwent thyroid surgery between October 2012 and August 2015. Intact parathyroid hormone (iPTH) and serum calcium (sCa) were assessed in all patients. iPTH (normal range, 15–65 pg/ml) was assessed using a commercially available iPTH assay (Elecsys 1010; Roche Diagnostics, Mannheim, Germany). sCa (normal range, 2.00–3.00 mmol/L) was measured on a conventional standard autoanalyzer. The diagnosis of hypoparathyroidism was established based on the iPTH and sCa on the morning of postoperative day 1 (12–24 hours postoperatively). Within this cohort, iPTH and sCa on the morning of postoperative day 1 of all patients were below the lower limit of the normal range. The patients were diagnosed with benign or malignant thyroid nodules. The patients with a diagnosis of primary parathyroid diseases or laryngeal cancer with thyroidectomy were excluded.

Patient data regarding the surgical procedure, the change in parathyroid function, and the management of hypoparathyroidism were reviewed. To patients with benign thyroid nodules >4 cm, lobectomy or total/near-total thyroidectomy was offered; to patients with malignant thyroid nodules, lobectomy/TT alone or lobectomy/TT with central-compartment (level VI) neck dissection was offered. Types of reoperation included lobectomy/TT alone, lobectomy/TT with CND, or unilateral/bilateral CND alone. All surgeries were performed by experienced surgeons with high-volume surgeries (more than 100 cases/year).

Long-term biochemical follow-up tests were performed 1, 6, and 12 months postoperatively. The diagnosis of permanent hypoparathyroidism required that features of hypoparathyroidism have persisted for at least 6 months after surgery (9).

Prophylactic intravenous calcium was routinely administered after bilateral thyroid surgery. Usually, 1 to 3 g of elemental calcium with active vitamin D3 and calcitriol orally divided was administered if the iPTH on the morning of postoperative day 1 was <15 pg/ml. The doses of calcium and active vitamin D3 would be increased when hypoparathyroid patients also had malabsorption syndrome. Calcium supplementation was stopped if the iPTH level exceeded 15 pg/ml no matter what the sCa level was.

### Statistical analyses

All data were collected for statistical analysis using the SPSS 17.0 software (SPSS Inc., Chicago, IL, USA). The unifactorial analysis of variance (ANOVA) and chi-square test were used to examine the difference between groups. The logistic regression analysis model was fitted for transient and permanent hypoparathyroidism. The level of significance was set at 0.05.

## Results

### Incidence rate of postoperative hypoparathyroidism

This retrospective analysis enrolled 2,903 patients who had thyroid surgery. Within this cohort, 637 patients (mean age 42.5 years; range 9–82) developed postoperative hypoparathyroidism, and the incidence rate of postoperative hypoparathyroidism was 21.94% (637/2,903). A total of 333 patients developed transient hypoparathyroidism, and the incidence rate of transient hypoparathyroidism was 11.47% (333/2,903). In addition, 304 developed permanent hypoparathyroidism, and the incidence rate of permanent hypoparathyroidism was 10.47% (304/2,903). The incidence rates of postoperative hypoparathyroidism under the clinicopathological characteristics of patients who had thyroid surgery are summarized in **Table 1**. The differences between incidence rates of transient and permanent hypoparathyroidism under several pathologic types and surgical procedures were statistically significant (p < 0.001). The main characteristics of the patients with postoperative hypoparathyroidism are summarized in **Table 2**.

**Table 1:** The incidence rate of postoperative hypoparathyroidism under the clinicopathological characteristics of patients who had thyroid surgery (*n* = 2,903).

Characteristic	No. of patients (*n* = 2,903)	Incidence rate of transient HypoPT		Incidence rate of permanent HypoPT		Total	
		No. of patients (*n* = 333)	%	No. of patients (*n* = 304)	%	No. of patients (*n* = 637)	%
Pathologic type							
Benign	974	30	3.08	20	2.05	50	5.13
Malignant	1,929	303	15.71	284	14.72	587	30.43
Surgical Procedure							
Lobectomy	186	3	1.61	3	1.61	6	3.22
TT alone	360	35	9.72	44	12.22	79	21.94
Lobectomy+CND	1,254	11	0.88	3	0.24	14	1.12
TT+CND	1,103	284	25.75	254	23.03	538	48.78

HypoPT, hypoparathyroidism; TT, total thyroidectomy; CND, central-compartment neck dissection.

**Table 2:** Characteristics of patients with postoperative hypoparathyroidism (*n* = 637).

Characteristic	No. of patients	%
Age (years)		
<45	362	56.83
≥45	275	43.17
Gender		
Male	193	30.30
Female	444	69.70
Pathologic type		
Benign	50	7.85
Malignant	587	92.15
Surgical procedure		
Lobectomy	6	0.94
TT alone	79	12.40
Lobectomy+CND	14	2.20
TT+CND	538	84.46
TT		
Yes	617	96.86
No	20	3.14
CND		
Yes	552	88.66
No	85	13.34
Reoperation		
Yes	77	12.09
No	560	87.91
Incidental parathyroidectomy		
Yes	224	35.16
No	413	64.84
Symptom		
None	461	72.37
Numbness	130	20.41
Twitch	46	7.22

TT, total thyroidectomy; CND, central-compartment neck dissection.

### Measurement of iPTH

iPTH on the morning of postoperative day 1 of all patients ranged from 1.20 to 14.98 pg/ml (mean ± SD, 7.34 ± 3.89 pg/ml). The differences in iPTH in various diagnoses and surgical procedures were statistically significant (p < 0.001). The iPTH was lower in patients with malignant thyroid nodules or who underwent TT and CND. The difference in iPTH between the TT+CND group and TT alone group was statistically significant (p = 0.001), but the difference was not statistically significant (p = 0.823) between the lobectomy+CND group and the lobectomy group. The study revealed that reoperation was not associated with iPTH (p = 0.538). The differences in iPTH under clinicopathological characteristics are summarized in **Table 3**.

**Table 3:** The difference in iPTH under their clinicopathological characteristics.

Characteristic	iPTH (pg/ml)	p
**Age (years)**		0.181
<45	7.18 ± 3.84	
≥45	7.54 ± 3.94	
**Gender**		0.608
Male	7.46 ± 3.99	
Female	7.31 ± 3.83	
**Pathologic type**		0.000
Benign	8.97 ± 4.09	
Malignant	7.19 ± 3.82	
**Surgical procedure**		0.000
Lobectomy	11.12 ± 4.19	
TT alone	8.20 ± 4.01	
Lobectomy+CND	10.85 ± 3.57	
TT+CND	6.92 ± 3.70	
**TT**		0.000
Yes	7.11 ± 3.77	
No	10.91 ± 3.67	
**CND**		0.000
Yes	7.14 ± 3.81	
No	8.47 ± 4.10	
**Reoperation**		0.538
Yes	7.58 ± 4.02	
No	7.33 ± 3.86	
Incidental parathyroidectomy		0.296
Yes	3.38 ± 0.78	
No	3.75 ± 1.55	

TT, total thyroidectomy; CND, central-compartment neck dissection; iPTH, intact parathyroid hormone.

The difference in iPTH between transient hypoparathyroidism and permanent hypoparathyroidism was statistically significant (p < 0.001) on the morning of postoperative day 1. The iPTH was higher in patients whose parathyroid function returned to normal (8.34 ± 3.95 vs. 6.08 ± 3.39 pg/ml). The iPTH on the morning of postoperative day 1 of transient and permanent hypoparathyroidism is summarized in **Table 4**.

**Table 4:** The iPTH on the morning of postoperative day 1 of transient and permanent hypoparathyroidism (*n* = 637).

iPTH (pg/ml)	Transient (*n* = 333)		Permanent (*n* = 304)	
	No. of patients	%	No. of patients	%
**<5**	102	30.63	159	52.30
**5–9.99**	129	38.74	104	34.21
**10–14.99**	102	30.63	41	13.49

iPTH, intact parathyroid hormone.

### Measurement of sCa

The values of sCa ranged from 1.00 to 1.99 mmol/L (mean ± SD, 1.81 ± 0.17 mmol/L). Hypocalcemia led to numbness in the hands or feet of 130 patients (20.41%, 130/637) and twitching in the hands or feet of 46 patients (7.22%, 46/637), and 461 patients (72.37%, 461/637) had no signs and symptoms. The difference in sCa between patients with different symptoms was statistically significant (p < 0.001). The value of sCa was 1.80 ± 0.18 mmol/L in the no-symptom group, 1.77 ± 0.16 mmol/L in the numbness group, and 1.81 ± 0.15 mmol/L in the twitch group.

### Recovery rate of parathyroid function

The parathyroid function of 52.28% (333/637) patients was restored. In the logistic regression analysis model, the pathologic type (p = 0.000), TT (p = 0.026), and CND (p = 0.022) were independently associated with the recovery of parathyroid function. Patients who were diagnosed with malignant thyroid nodules or had undergone TT and CND had higher risks of developing permanent hypoparathyroidism than others. No association was observed in other factors including age, gender, reoperation, or incidental parathyroidectomy. The recovery rates of parathyroid function according to the clinicopathological characteristics of patients with postoperative hypoparathyroidism are summarized in **Table 5**.

**Table 5:** The recovery rate of parathyroid function under the clinicopathological characteristics of patients with postoperative hypoparathyroidism (*n* = 637).

Characteristic	Transient (*n* = 333)	Permanent (*n* = 304)	Recovery rate of parathyroid function (%)	p	OR	95% CI
**Age (years)**				0.640	1.07	0.81–1.42
<45	188	174	51.93			
≥45	145	130	52.73			
**Gender**				0.349	1.15	0.86–1.55
Male	67	72	48.20			
Female	234	210	52.70			
**Pathologic type**				0.000	0.29	0.14–0.57
Benign	30	20	60			
Malignant	303	284	51.62			
**TT**				0.028	0.50	0.27–0.93
Yes	319	298	51.70			
No	14	6	70			
**CND**				0.023	0.894	0.61–1.31
Yes	295	357	45.25			
No	38	47	44.71			
**Reoperation**				0.069	0.68	0.44–1.03
Yes	33	44	42.86			
No	305	255	54.46			
**Incidental parathyroidectomy**				0.881	1.025	0.74–1.42
Yes	118	106	52.68			
No	215	198	52.06			

TT, total thyroidectomy; CND, central-compartment neck dissection.

### Rate of resection of parathyroid

It was confirmed that parathyroid glands in the thyroid gland and CND specimens were excised from 224 (35.16%, 224/637) patients with postoperative hypoparathyroidism by postoperative pathology. The rate of inadvertent parathyroid gland removal after a thyroid operation was 7.72% (224/2903). There was no correlation between the parathyroid gland removal and the recovery of parathyroid function independently (p = 0.881). In the logistic regression analysis model, gender (p = 0.005) and CND (p = 0.002) were independently associated with the resection of parathyroid. Female patients who underwent CND had a higher risk of resection of parathyroid than others. No associations were observed in other factors including age, pathologic type, TT, or reoperation. The rate of resection of parathyroid under the clinicopathological characteristics of patients with postoperative hypoparathyroidism is summarized in **Table 6**.

**Table 6:** The rate of incidental parathyroidectomy under the clinicopathological characteristics of patients with postoperative hypoparathyroidism (*n* = 637).

Characteristic	Incidental parathyroidectomy		Rate of incidental parathyroidectomy (%)	p	OR	95%CI
	Yes (*n* = 224)	No (*n* =413)				
**Age (years)**				0.298	0.99	0.98–1.01
<45	138	224	38.12			
≥45	86	189	31.28			
**Gender**				0.005	1.60	1.16–2.22
Male	55	138	28.50			
Female	169	275	38.06			
**Pathologic type**				0.303	1.58	0.66–3.79
Benign	6	44	12			
Malignant	218	369	37.14			
**TT**				0.987	1.01	0.40–2.56
Yes	217	400	35.17			
No	7	13	35			
**CND**				0.002	2.91	1.50–5.67
Yes	211	341	38.22			
No	13	72	15.29			
**Reoperation**				0.063	0.64	0.40–1.03
Yes	22	55	28.57			
No	202	358	36.07			

TT, total thyroidectomy; CND, central-compartment neck dissection.

### Permanent postoperative hypoparathyroidism evaluation

The PPHE was established on the risk factors and clinical practice. The iPTH, sCa, surgical procedure, reoperation, and pathologic type were selected as the reference indexes for PPHE. The scores of each reference index are shown as follows:

iPTH (pg/ml): {10.00–14.99} = 1, {5.00–9.99} = 2, {<5.00} = 3;
sCa (mmol/L): {1.75–1.99} = 1, {1.50–1.74} = 2, {<1.50} = 3;
Surgical procedure: {lobectomy} = 1, {lobectomy+CND} = 2, {TT} = 3, {TT+CND} = 4;
Reoperation: {no} = 0, {yes} = 1;
Pathologic type: {benign} = 1, {malignant} = 2.

The formula for PPHE is as follows: {iPTH} + {sCa} + {surgical procedure} + {reoperation} + {pathologic type}. The scoring system was developed, and we scored low, middle, and high risk of permanent postoperative hypoparathyroidism as 4–6, 7–9, and 10–13, respectively.

The scores for PPHE of all patients ranged from 4 to 13 (mean ± SD, 9.28 ± 1.64). The recovery rate of parathyroid function was 75% (18/24) in the low-risk group, 61.27% (193/315) in the middle-risk group, and 40.94% (122/298) in the high-risk group. The differences in the recovery rates of parathyroid function in distinct risk groups were statistically significant (p < 0.001).

## Discussion

Postoperative hypoparathyroidism caused by temporarily “disturbed” parathyroid metabolism is a clinically relevant complication that can occur after thyroid surgery. It usually is due to inadvertent or unavoidable removal of or damage to the parathyroid glands and/or their blood supply. Earlier reports had suggested that after thyroid surgery, transient hypoparathyroidism range from 6.9% to 53.6% (10–13), and permanent hypoparathyroidism range from 0.4% to 33% (5, 12, 14–16). In our study, the incidence rate of transient hypoparathyroidism was 11.47%, and the incidence rate of permanent hypoparathyroidism was 10.47%.

### Pathologic type and postoperative hypoparathyroidism

Of the 637 patients with postoperative hypoparathyroidism, 92.15% were diagnosed with malignant thyroid nodules. However, the patients were treated in local clinics instead of our center when they were diagnosed with benign thyroid nodules. These may be the main reason for the low constituent ratio of benign thyroid nodules to malignant ones in our study.

### Surgical procedure and postoperative hypoparathyroidism

Bilateral thyroid surgery is a major risk factor for both transient and permanent hypoparathyroidism (5–7). The results of our study were consistent with the findings of others. The iPTH on the morning of postoperative day 1 and recovery rate of parathyroid function were lower than in patients with unilateral (lobectomy ± CND) thyroid surgery. These can be explained by the fact that bilateral thyroid surgery may increase the risk of traumatic or ischemic damage to parathyroid glands caused by surgical manipulation on both sides (17). However, postoperative hypoparathyroidism after unilateral thyroid surgery had little been reported in the literature. The incidence rates of transient and permanent hypoparathyroidism of unilateral thyroid surgery were 0.97% (14/1440) and 0.42% (6/1440), respectively. In the 20 patients with unilateral thyroid surgery, the iPTH of six patients was under 10 pg/ml. This may be because some people only have two parathyroid glands.

The role of central-compartment neck dissection in the treatment of cN0 papillary thyroid carcinoma is debatable. It has been repeatedly emphasized that the procedure increases the risk of parathyroid-related complications, with rates of permanent hypoparathyroidism of 4%–11% (18–21). As previously reported, rates of transient hypoparathyroidism were also higher in patients who had CND compared to patients who had TT alone (21–27). In our cohort, the difference in iPTH between the TT+CND group and TT alone group was statistically significant (p = 0.001), and undergoing CND in the same period was independently associated with the recovery of parathyroid function (p = 0.023, OR = 0.894) and the resection of parathyroid (p = 0.002, OR = 2.91). It also may be because that normal parathyroid tissue has an appearance similar to that of enlarged lymph nodes, and another reason may be due to injury of vascular pedicles when parathyroid glands share the same blood supply as the thyroid (28).

### Reoperation and postoperative hypoparathyroidism

Patients who have undergone primary surgery for either benign or malignant disease may subsequently be indicated for a further thyroid procedure. Removal of all or part of the gland at the primary procedure leads to gross anatomical disruption. Further disruption results from postoperative tissue change through fibrosis and scarring (29). Combined, these effects may account for the higher rates of complications seen in previous case series of reoperative thyroid surgery (30–34). However, the difference between the primary surgery group and the reoperation group was not statistically significant within our cohort. It may be because patients have sufficient time to recover their blood supply to the parathyroid gland by collateral regeneration of the vessels from relatively undamaged surrounding tissues and scar tissue of the previous surgery, and the parathyroid gland may be more resistant to ischemic injury in a reoperative procedure even though fibrotic scar tissue makes sharp dissection difficult.

### Prevention of hypoparathyroidism

In our study, the rate of resection of parathyroid was only 35.16% (224/637) in the patients with postoperative hypoparathyroidism. The other two-thirds of postoperative hypoparathyroidism might be due to damage to the blood supply of parathyroid glands. Identification and preservation of the parathyroid glands *in situ* and avoidance of injury to their vascular pedicles were key factors in managing hypoparathyroidism. Rapid intraoperative parathyroid hormone (rIO-PTH) assay levels were used to identify parathyroids as a parameter in thyroid surgery (35, 36). Carbon nanoparticles (CNs) can be injected as a suspension (China Food and Drug Administration approval H20041829; Lai Mei Pharmaceutical Co., Chongqing, China) that can enter the lymphatic capillaries. After the injection of CN suspension into thyroid parenchyma, the CNs will penetrate and diffuse the whole thyroid lobe, enter the lymphatic vessels rapidly, accumulate in the lymph, and stain them back thereby. However, the parathyroid glands have no lymph–vessel connections to the thyroid to be stained black. CN suspension injection has been applied recently to help identify parathyroids during thyroid surgery (37–40). Angiography with the fluorescent dye indocyanine green (ICG) was performed to visualize the vascularization of identified parathyroid glands (41). Every effort must thus be made to preserve vascular supply to the superior parathyroid gland. However, it is often difficult to do so for the inferior parathyroid gland, as it is more prone to ischemic injury during CND.

Under the premise that it was confirmed on intraoperative frozen-section pathology to avoid autograft of a lymph node metastasis, any parathyroid gland that looks likely to be totally devascularized can be removed during the operation and autotransplanted into well-vascularized muscles such as the sternocleidomastoid muscle or the forearm muscles (42). The thyroid gland and CND specimens excised should also be examined carefully to discover any inadvertently removed parathyroid glands, and these should be autotransplanted (43).

### Permanent postoperative hypoparathyroidism evaluation

It is a very significant topic in clinical research to evaluate the surgical risk level of patients objectively. Glasgow coma scale (GCS), acute physiology and chronic health evaluation II (APACHE II), and physiological and operative score for the enumeration of mortality and morbidity (POSSUM) are widely used in the prognosis evaluation of surgical patients. However, there are few scoring models for the incidence of postoperative complications in patients with thyroid nodules.

According to the results of multivariate analysis, we established the risk function of permanent postoperative hypoparathyroidism and used the ROC curve test to evaluate the efficacy. The area under the ROC curve was 0.7, and the efficacy evaluation was moderate. We thought that it might be limited to evaluating permanent postoperative hypoparathyroidism only by risk factors. Therefore, according to the characteristics of thyroid surgery, combined with the results of multivariate analysis and clinical practice, iPTH, sCa, surgical procedure, reoperation, and pathologic type were selected as the reference indexes for PPHE. We distributed the score into the low, middle, and high risks to evaluate permanent postoperative hypoparathyroidism. The differences in the recovery rates of parathyroid function in distinct risk groups were statistically significant (p < 0.001).

PPHE can forecast the risk of permanent postoperative hypoparathyroidism well. According to it, the patients can be monitored and treated individually, which has important clinical application value. Prospective studies that are multicenter and larger are required to continuously improve PPHE.

## Conclusions

In conclusion, hypoparathyroidism occurs due to thyroid surgery, especially in patients with malignant thyroid nodules. In patients with bilateral thyroid surgery and CND in the same period, there are more postoperative hypoparathyroidism and more resection of the parathyroid. The reoperation for recurrence is not associated with hypoparathyroidism. Identification and preservation of the parathyroid glands *in situ* and avoidance of injury to their vascular pedicles are key factors in managing hypoparathyroidism. PPHE can forecast the risk of permanent postoperative hypoparathyroidism well.

## Data availability statement

The original contributions presented in the study are included in the article/**Supplementary Material**. Further inquiries can be directed to the corresponding author.

## Ethics statement

Ethical review and approval were not required for the study on human participants in accordance with the local legislation and institutional requirements. Written informed consent from the patients was not required to participate in this study in accordance with national legislation and institutional requirements.

## Author contributions 

XW performed the methodology, data analysis, and writing. SW performed the formal analysis and writing. YC and ZG performed the validation. CL and WH performed the project administration. All authors contributed to the article and approved the submitted version.
